# Psilocybin effects on brain functional connectivity: a systematic review of fMRI studies

**DOI:** 10.1007/s44192-026-00384-w

**Published:** 2026-03-19

**Authors:** Àlvar Farré-Colomés, Olga Rublinetska, Óscar Soto-Angona

**Affiliations:** 1 Department of Addictive Behaviour and Addiction Medicine, Central Institute of Mental Health, Medical Faculty of Mannheim, Heidelberg University, J 5, 68159 Mannheim, Germany; 2 Sociedad Española de Medicina Psicodélica (SEMPsi), Barcelona, Spain; 3 ANIMA Group, Institut de Recerca Sant Joan de Déu (IRSJD), Santa Rosa 39-57, 08950 Esplugues de Llobregat, Spain; 4 Parc Sanitari Sant Joan de Déu, Doctor Antoni Pujadas 42, 08830 Sant Boi de Llobregat, Spain

**Keywords:** Psilocybin, FMRI, Functional connectivity, Depression, ACC, Amygdala

## Abstract

Psilocybin-assisted therapies are innovative therapeutic approaches, particularly in the treatment of depression. However, there are sparse studies providing functional magnetic resonance imaging (fMRI) evidence elucidating the underlying biological mechanisms that support clinical outcomes. This review aims to comprehensively gather all the evidence reported in psilocybin studies using fMRI techniques. Independent extraction of articles was conducted by 2 authors using predefined data fields. 20 unique datasets were identified, with 5 including participants diagnosed with depression. Dropout rates were found to be high, and follow-up scanning timepoints were lacking in most of the studies. Most research has focused on the amygdala, the anterior cingulate cortex and the prefrontal cortex, as key regions involved in the effects of psilocybin. However, the current literature exhibits inconsistency in methods and designs. Further research is necessary to better define psilocybin’s impact on the human brain and its potential to enhance psychotherapy outcomes.

## Introduction

Psychedelics have been the object of scientific research for many years, with increasing medical interest in the last decade. Classical psychedelic drugs, such as mescaline, LSD and N,N-Dimethyltryptamine (DMT), are defined as substances that primarily act as agonists of the serotonin 5-hydroxy-tryptamine type 2 A (5-HT_2A_) receptors [1]. The pharmacology and pharmacodynamics of these substances have been extensively studied, particularly during the 1960s. Currently, their therapeutic potential in psychiatry has brought them back into focus, and numerous investigations have emerged with the purpose of demonstrating the safety, efficacy, and non-addictive nature of their active compounds.

Psilocybin (Psi) is one of the most investigated psychedelics and occurs naturally in the almost 200 psychoactive genera of mushrooms [2]. Over the years, multiple studies have suggested its therapeutic potential in treating a wide variety of conditions, including cluster headaches, anorexia, end-of-life anxiety, treatment-resistant depression, nicotine dependence, alcohol use disorder and more [3–8].

In the human body, Psi is rapidly dephosphorylated into its active metabolite, psilocin, which then acts as a non-selective agonist of numerous serotonin receptors, primarily serotonin 2 A receptors (5-HT_2A_R) [9–11]. These receptors are distributed across cortical and subcortical structures of the brain, with the Prefrontal Cortex (PFC) showing the strongest correlation between receptor occupancy and subjective Psi effects [12]. However, it has been suggested that other serotonin receptors, including the 5-HT_1A_R, may contribute to the visual and attention-disrupting effects of Psi [13, 14]. The intricate modulation at the neuronal level resulting from the activation of different serotonin receptors after Psi intake has been extensively described by Vollenweider and Preller [15] and supported by neuroimaging studies, demonstrating the complexity of Psi effects in the human brain [16]. It is important to note that regions with high densities of 5-HT_2A_R are not the only areas responding to Psi. As a result, predicting which brain regions will exhibit activity changes in response to Psi is challenging, making it difficult to directly correlate alterations in brain connectivity to clinical outcomes in the treatment of psychiatric disorders.

Despite the recent surge of interest in Psi as a psychotherapeutic tool, the mechanisms of action underlying its therapeutic effects are still largely under debate. Several models have been proposed to explain the neurobiological underpinnings of psychedelics, among them the Relaxed Beliefs Under Psychedelics (REBUS) and Brain Entropy model, the Cortico-Striatal-Thalamo-Cortical Model (CSTC), and the Cortico-Claustro-Cortical (CCC) Model [17]. The REBUS model postulates that psychedelics have an entropic effect on cortical activity, leading to a disruption in top-down cognitive processing and increased neural flexibility [16]. The CSTC model, in contrast, suggests that 5-HT_2A_R activation increases bottom-up informational flow by reducing thalamic sensory gating [18]. Finally, according to the CCC model, psychedelic effects stem from receptor activation within the claustrum, leading to disruptions in higher-order cortical networks mediated by CCC circuits that underpin the subjective psychedelic experience [17].

Understanding how Psi influences neural circuits could inform the development of strategies to enhance its therapeutic effects, minimize potential risks, and uncover new insights into the functions of these circuits in both health and disease. However, there remains a substantial absence of neuroimaging evidence necessary to construct a comprehensive and precise understanding of the therapeutic potential of Psi. The majority of recent studies focus primarily on the clinical outcomes of their interventions, lacking neurophysiological support for their findings. Over a 10-year period (from 2010 to 2019), only 14 articles were published investigating functional connectivity (FC) changes related to Psi, with just 7 of these presenting new data and the rest analyzing previously published datasets. However, from 2020 to the present, 15 articles have been published on the topic, with 10 of them providing new datasets. There is therefore a remarkable and growing interest in Psi effects on brain FC.

While numerous reviews, including systematic reviews, have examined psychedelic-assisted psychotherapy treatment, they predominantly focus on clinical outcomes and therapeutic effects in psychiatric disorders, typically assessed via self-reported and clinician-administered symptom rating scales [4, 19, 20]. Only two reviews about neuroimaging and psychedelics were identified, one that focuses exclusively on resting-state connectivity [21], while the other covers all neuroimaging techniques [22]. Both examine psychedelics broadly, encompassing various compounds in their analyses. Consequently, we decided to focus on the first-in-class psychedelic compound (Psi) and review all fMRI studies analyzing its effects on brain connectivity. To date, no other review has detailed the effects of Psi on FC in the human brain.

The purpose of this systematic review is to provide a comprehensive overview of the functional connectivity changes underlying both the acute and long-term effects of Psi on the brain. By elucidating its neurobiological foundations, we aim to highlight the possibility for a reliable imaging biomarker of its therapeutic effects and to discuss the existing evidence for the aforementioned theoretical models. We also intend to highlight the importance of generating new neuroimaging datasets for future analyses and encourage the inclusion of fMRI assessments in clinical trials, including throughout follow-up periods.

## Materials and methods

The present study adhered to the standardized PRISMA guidelines [23, 24], although it did not have a previous protocol registration. Due to the limited number of available datasets, no restrictions were applied regarding participant characteristics, publication year, or intervention type, as the small and methodologically diverse sample of studies made such distinctions impractical. Only articles published in peer-reviewed journals in English and Spanish up to the time of the last search were considered for inclusion in the review.

The search process involved three independent academic platforms (PubMed, ScienceOpen, Web of Science) and was finalized on March 13th, 2025. This review includes all the clinical trials and original research studies investigating the effects of Psi on brain FC using functional MRI, published between February 2012 and July 2024. The script used for the search was:

(Psilocybin[Title/Abstract]) AND ((fMRI[Title/Abstract]) OR (functional MRI[Title/Abstract]) OR (functional connectivity[Title/Abstract])).

**Fig. 1:**
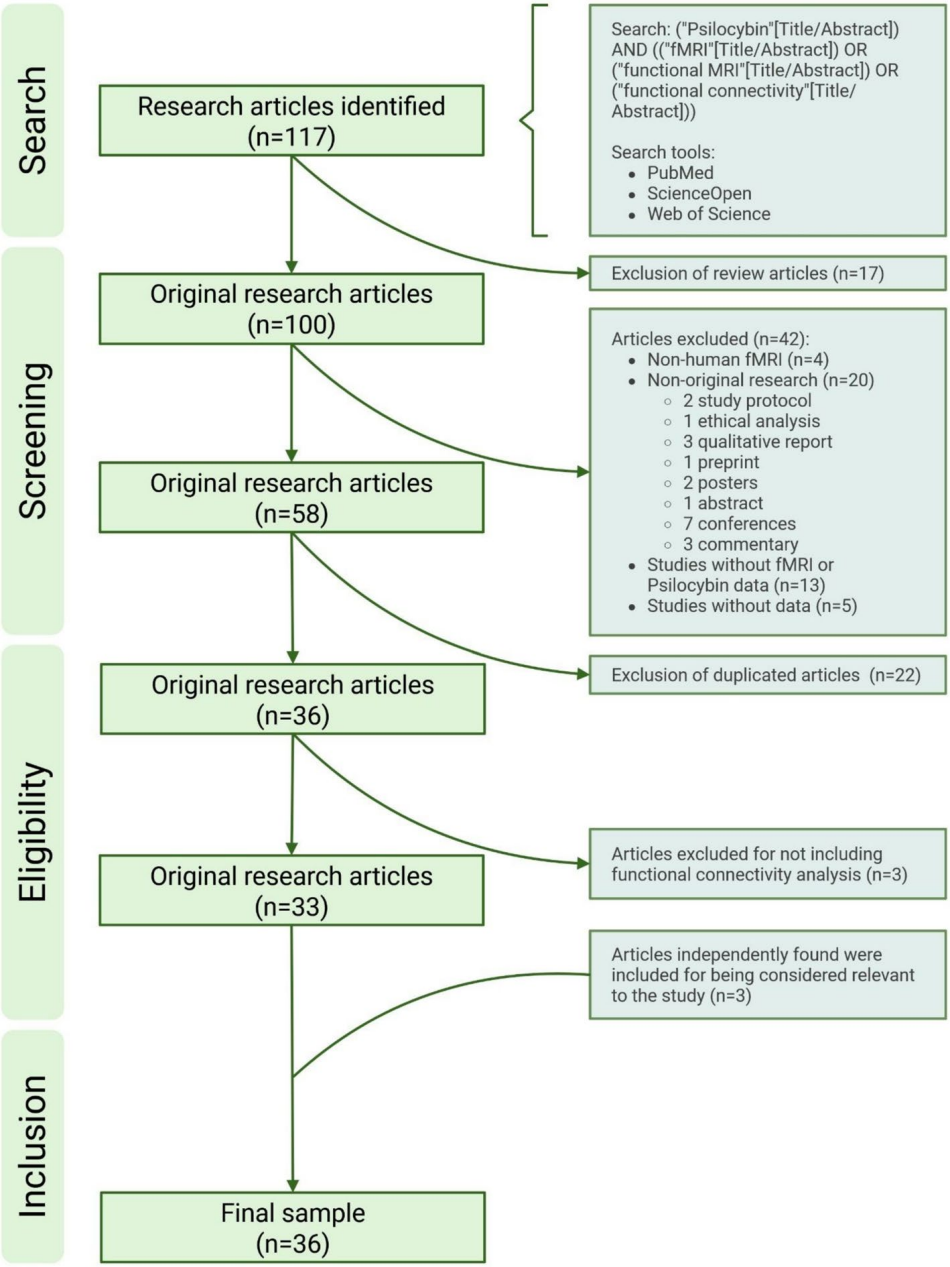
Flow Chart—Diagram displaying the article search and selection process with the number of articles added or omitted in each step

The entire screening process is illustrated in Fig. 1. The final number of research articles included was 100, after the exclusion of reviews. From that sample, 42 articles were excluded for not meeting the inclusion criteria. Consequently, the total number of articles was reduced to 58 articles, and after eliminating duplicates, 36 articles remained. After revision of all 36 articles, 3 were found unsuitable for the review due to a lack of FC data analysis. Additionally, 3 articles that were not found during the search but were mentioned in the studied articles were considered to meet the inclusion criteria and therefore included in the study.

The selected articles underwent a comprehensive review, with information independently extracted by 2 reviewers. Each reviewer contributed their collected information, which was subsequently combined for discussion and analysis. The database was structured to allow differentiation between article types, procedures, participants, and other key features. In case of major uncertainties, corresponding authors were contacted for clarification.

The data summary encompassed various items, with key components being the sample size, subject diagnosis (healthy or otherwise), the timing of Psi administration relative to scanning, the presence of a follow-up scan, the Regions of Interest (ROIs) used for the connectivity analyses, and the scales employed to assess clinical effects. Some items were added during the review process upon recognizing their significance, including the use of placebo, participants’ prior experience with psychedelics, and the Psi dose and number of administrations. These additions were crucial to understanding the study designs, interpreting the results, and facilitating comparisons between studies.

All included studies adhered to proper principles to mitigate bias. For example, all studies reported the initial and final number of participants, employed blind study designs, or provided justification otherwise. In cases where participants were not healthy volunteers, the studies consistently followed the intention-to-treat principles.

## Results

A final sample of 36 articles was used to conduct this review. Among these, 20 contain original datasets (see Table 1).

**Table 1:** Number of original datasets used in posterior studies. There is a total of 20 original datasets, and 17 studies that have included analysis from previously published datasets

Original data		Carhart-Harris [79]	Roseman [85]	Tagliazucchi [40]	Kraehenmann [41]	Lebedev [32]	Grimm [53]	Lord [33]	Varley (2020)	Mertens [74]	Daws [30] (1)*	Girn [34]	Olsen [35]	Tolle [73]	Wall [77]	Copa [86]	Mortaheb (2024)	**S**troliker (2024)	Repetitions
1	Carhart-Harris [39]	X	X	X		X		X	X			X							7
2	Carhart-Harris [39]																		0
3	Kraehenmann [41]				X		X												2
4	Preller [31]																		0
5	Carhart-Harris [28]										X				X	X			3
6	Roseman [29]									X									1
7	Smigielski [27]																		0
8	Preller [88]																	X	1
9	Barrett [25]																		0
10	Barrett [44]																		0
11	Mason [42]													X			X		2
12	Madsen [43]												X						1
13	Duerler [80]																		0
14	Doss [18]																		0
15	McCulloch [21]																		0
16	Daws [30] (2)*																		0
17	Gaddis [26]																		0
18	Shukuroglou [105]																		0
19	Madsen [43]																		0
20	Siegel [45]																		0
	Total repeated datasets	17																	

*Daws et al. [30] includes an original dataset (DB-RCT) and a previously published dataset (Open Label Trial, from Carhart-Harris et al. [28])

### Participant characteristics

The studies include a total of 364 participants, with sample sizes ranging from 10 to 38 participants. Of these, 211 were male and 153 were female. The average age of participants was 37.28, with a standard deviation of 8.4. Additional information on participant demographics and experimental design is provided in Table 2.

**Table 2:** Demographic data of the participants from the original datasets used in the articles included in the review

Source	Sample size (n)	Diagnosis	Mean age (SD)	Male–female %	Previous Psi use
Carhart-Harris et al. [39]	15	HC	32 (8.9)	86.6%–13.4%	All, but not within 6 weeks of study
Carhart-Harris et al. [39]	10	HC	31 (7.5)	90%–10%	All, but not within 6 weeks of study
Kraehenmann et al. [41]	25	HC	24.2 (3.42)	64%–36%	28%, but not within 24.6 ± 31.2 months of study
Preller et al. [31]	21	HC	26.48 (4.76)	57.14%–42.86%	ns
Carhart-Harris et al. [28]	15	TRMD	42.8 (10.5)	68.75%–31.25%	25%, frequency or length of time prior to start of study unspecified
Roseman et al. [29]	19	TRMD	44.7 (10.9)	68.42%–31.58%	ns
Smigielski et al. [27]	38	HC–EM	51.66 (8.32)	60.52%–39.48%	47%, 26% consumed ≤ 3 × over 20 yrs ago; 21% consumed ≤ 3 times during last 20 years
Preller et al. [88]	23	HC	26.3 (20–40)	52.17%–47.83%	35%, but not within 2wks of study
Barrett et al. [25]	15	HC–EM	51.3 (12.3)	41.6%–58.4%	All with 1–4 uses during lifetime, average most recent use = 8.3 years ago
Barrett et al. [44]	12	HC	32.1 (7.5)	66.6%–33.4%	59%, but not within 2 months of this study (use in previous study) and not within 5 years before that
Mason et al. [42]	48	HC	22.73 (2.9)	60%–40%	All, but not within 3 months of study
Madsen et al. [43]	15	HC	34.3 (9.8)	60%–40%	53% w/limited use: all but 1 subject w/1 use during their lifetime and none in year before study
Duerler et al. [80]	15	HC	26.86 (ns)	66.6%–33.4%	ns
Doss et al. [18]	24	MDD	39.83 (12.23)	33.3%–66.7%	ns, but participants with > 10 uses during lifetime + use in past 6 months excluded from study
McCulloch et al. [21]	10	HC	28.3 (3.4)	60%–40%	None
Daws et al. [30] (2)	22	MDD	44.5 (11)	63.63%–36.37%	55%, frequency or length of time prior to start of study unspecified
Gaddis et al. [26]^†^	38	HC–EM	56 (ns)	60%–40%	All, but not within 2 months of study, 50% had previous use but not within 5 years prior
Shukuroglou et al. [105]*	19	TRMD	43.1 (10.5)	68.42%–31.58%	ns
Madsen et al. [43]	10	CCH	49.4 (12.9)	50%–50%	ns
Siegel et al. [45]	7	HC	36–45 (ns)	57.14%–42.86%	All, minimum 6 months prior the study

^†^, Sample from Barrett et al. [25]; *, Sample from Carhart-Harris et al. [28]; HC, Healthy controls; EM, Experienced meditators; TRMD, Treatment resistant major depression; MDD, Major depressive disorder; CCH, Chronic cluster headache; ns, not specified

Of the 20 unique datasets, 15 involved healthy participants. Subjects were evaluated based on their medical history, physical examination, blood analyses, and electrocardiography, along with a structured clinical interview to confirm psychiatrically health. Common exclusion criteria included past or present psychiatric disorders, a history of major psychiatric disorders in first-degree relatives, current or recent history of substance dependence, cardiovascular disease, history of head injury, claustrophobia, and previous adverse reactions to hallucinogenic drugs. The history of psychedelic use varied among the studies (see Table 1).

Three of the original datasets evaluated the effects of Psi in experienced meditators [25–27]. Two datasets included participants with treatment-resistant major depression (TRMD) [28, 29], while two others included patients with major depressive disorder (MDD) [18, 30]. Patients with TRMD had to have unsuccessfully tried two courses of different antidepressant treatments lasting at least 6 weeks within the current depressive episode [28]. Patients with MDD were rated based on the Hamilton Depression Rating scale [HAM-D] or a modified version with scores of 16–17+. Exclusion criteria included alternative current or previously diagnosed psychiatric disorder and/or immediate (1st degree) family member with a diagnosed psychiatric disorder, among others.

### Study design

The majority of the studies used within-subjects and crossover designs (see Tables 3 and 4). Almost all the studies employing a crossover design incorporated a time interval, or wash-out period, of at least 14 days between sessions. An exception was Preller, Pokorny [31], in which a minimum of 10 days between dosing sessions was permitted. Among the 36 included studies, only 11 articles conducted task-based FC analyses, with the experimental tasks varying from amygdala reactivity to emotional discrimination tasks, among others (see Table 4).

**Table 3:** Summary of resting-state FC changes following psilocybin administration in healthy (n = 21 studies) and depressed patients (n = 5 studies). Includes study designs, analyzed brain networks/regions, and a summary of results

Psi dose/admin	Placebo	Effects/scan timepoints	Questionnaires	Data analysis	ROIs	Summary of findings
*Healthy participants*						
Carhart-Harris et al. [39]º*						
2 mg—IV	Vehicle (saline)	Single acute	SDI-VAS	ROI-to-ROI	ACC, PFC, PCC, Thalamus	↓ BOLD signal and CBF in thalamus, ACC and PCC ↓ BOLD and CBF in ACC/mPFC correlated positively with subjective effect ratings ↓ FC between mPFC & PCC
Carhart-Harris et al. [79]º*						
2 mg—IV	Vehicle (saline)	Single acute	SDI-VAS	SBC	Thalamus	↑ FC between DMN & TPN (DAN, SN, rFPN/ECN, AudN) ↑ Thalamocortical rsFC
Roseman et al. [85]º*						
2 mg—IV	Vehicle (saline)	Single acute	SDI-VAS	NetC	N/A	↑ FC: VisN-DAN, FPN, DMN; AudN-DMN, ECN, FPN, DAN; SMN-ECN, FPN, DAN; DMN-DAN, ECN, FPN; ECN-FPN; FPN-DAN ↓ FC between VisN & SMN
Tagliazucchi et al. [40]º*						
2 mg—IV	Vehicle (saline)	Single acute	None	DynC	ACC, PHG, Hippocampus	↑ FC within ACC and within Hippocampus ↑ FC between ACC and Hippocampus
Lebedev et al. [32]º*						
2 mg—IV	Vehicle (saline)	Single acute	BDI, STAI, SDI-VAS	Other	N/A	↓ FC between MTL & cortex, ↓ SN integrity and ↓ interhemispheric communication were associated with Psi-induced ego dissolution—correlation between intensity of ego dissolution and diversity of FC in anterior parahippocampal cortex
Smigielski et al. [27]^‡^						
0.315 mg/kg—Oral	Lactose	Pre (−4d) versus Post (+ 48 h)	5D-ASC, PEQ	ROI-to-ROI	PFC, PCC, Angular Gyrus	↓ FC between mPFC & PCC positively associated with ego dissolution state (measured by subjective self-boundlessness scores)
Lord et al. [33]º*						
2 mg—IV	Vehicle (saline)	Single acute	None	Other	N/A	↓ expression of BOLD phase-locking state overlapping with FPN (= ECN) ↑ global network coherence
Preller et al. [31]º*						
0.2 mg/kg—Oral	Mannitol	Three acute (+ 20 m, 40 m, 70 m)	5D-ASC	WBC	N/A	↓ FC in medial and lateral PFC, Cingulum, Insula, Temporoparietal junction ↑ FC in bilateral Occipital Cortex, Precuneus, rSTG, lPCG Baseline FC predicted Psi-induced FC changes
Varley et al. [106]º*						
2 mg—IV	Vehicle (saline)	Single acute	None	Other	N/A	↑ fractal dimension of cortical FC networks ↑ fractal dimension of BOLD time-series in DAN regions
Barrett et al. [25]º						
0.143 mg/kg—Oral	Low dose of Psi (unspecified)	Single acute	SDI-Verbal	NetC	Claustrum	↓ FC in right and left claustrum ↓ FC between right claustrum & DMN, AudN ↑ FC between right claustrum & FPN (= ECN); ↓FC between left claustrum & FPN (= ECN) Subjective effects of Psi predicted BOLD signal variance in claustrum
Mason et al. [42]^‡^						
0.17 mg/kg—Oral	Yes, unspecified	Single acute	5D-ASC, EDI, SWSL	NetC	N/A	↓ DMN FC predicted higher scores in acute & long-term creative thinking ↑ FC between DMN & FPN (= ECN) predicted decreases in acute & long-term creative thinking
Madsen et al. [43]º						
0.2 mg/kg (n = 4)/0.3 mg/kg (n = 11)—Oral	None	Four acute (+ 40 m, 80 m, 130 m, 300 m)	SDI-Verbal	NetC	N/A	- correlation between PPL & DMN, SN network integrity ↑ FC within DAN & ECN (= FPN) ↑ FC between DMN & SN, ECN (= FPN) ↑ FC between DAN & AudN, SMN
McCulloch et al. [21]º						
0.2 mg/kg (n = 4)/0.3 mg/kg (n = 6)—Oral	None	Pre (∼15d) versus Post (+ 1w & 3 m)	NEO-PI-R, MAAS, SDI, 11D-ASC, MEQ30, EDI, PEQ	NetC	N/A	↓ ECN FC 1w post-Psi admin, but ↓ was not significant at 3 m—correlation between ECN FC and mindfulness scores at 3 m ↓ 5-HT2AR levels at 1w correlated with ↓ ECN (= FPN) FC at 3 m
Gaddis et al. [26]º						
10 mg/70 kg (0.357 mg/kg)—Oral	Low dose of Psi (unspecified)	Single acute (+ 100 m)	MEQ, SDI-Verbal	SBC	Thalamus	↓ Intrathalamic FC ↓ Thalamocortical FC (Thalamus and DMN, VisN)
Girn et al. [34]º*						
2 mg—IV	Vehicle (saline)	Single acute	VAS, 11D-ASC	WBC	N/A	↓ Hierarchical organization in the cortex ↑ FC between association and sensorimotor cortical areas ↑ FC in SMN; ↓ FC in DMN; ↓ FC in DAN
Olsen et al. [35]º						
0.2 mg/kg (n = 4)/0.3 mg/kg (n = 11)—Oral	None	Four acute (+ 40 m, 80 m, 130 m, 300 m)	SDI-Verbal	DynC	N/A	- correlation between FC within FPN (= ECN) & subjective effect ratings and PPL ↓ FC between FPN (= ECN) and Cingulum, vmPFC, and parieto-occipital fissure
Tolle et al. [73]^‡^						
0.17 mg/kg—Oral	Vehicle (bitter lemon)	Single acute	11D-ASC	NetC	N/A	- correlation between Psi subjective effects and FC within the DMN and between DMN–Lymbic system Greater variability in FC between subjects under Psi effects is primarily in the DMN, while it is in the FPN (= ECN) under placebo
Madsen et al. [43]º						
0.14 mg/kg—Oral	Non	Pre (−1d) and post (+ 1w)	11D-ASC	NetC	Hypothalamus	↑ FC with the hypothalamus, thalamus and Caudate
Mortaheb et al. [107]^‡^						
0.17 mg/kg—Oral	Yes, unspecified	Single acute	5D-ASC, 11D-ASC	WBC	N/A	Generalized increase in whole-brain FC More integration and less segregation of networks under Psi
Siegel et al. [45]º*						
25 mg—Oral	Methylphenidate	Pre, acute (+ 60–180 min) and post (+ 3w)	MEQ30	WBC	N/A	↓ FC within networks and ↑ FC between networks Strongest changes in the DMN Persistent ↓ FC between the DMN and the anterior hippocampus + correlation with subjective psychedelic experience
Stroliker et al. [108]º*						
0.2 mg/kg—Oral	Mannitol	Acute (+ 70 m)	5D-ASC	NetC	VisN	↓ FC within the VisNet ↑ top-down inhibition + correlation with subjective effects
*TRMD/MDD patients*						
Carhart-Harris et al. [28]º						
1st: 10 mg 2nd: 25 mg—Oral	None	Pre versus Post (+ 24 h)	QIDS	SBC	Amygdala, ACC, PFC, PHG	↑ FC within DMN ↑ FC between ACC – PCC ↑ FC between vmPFC – IPC 24 h after Psi predicted treatment response at 5w ↓ FC between PHG – PFC 24 h after Psi predicted treatment response at 5w Amygdala FC not significantly altered post-Psi treatment
Doss et al. [18]º						
1st: 20 mg/70 kg (0.286 mg/kg) 2nd: 30 mg/70 kg (0.429 mg/kg) – Oral	None	Pre (−4w) versus Post (+ 1w)	GRID-HAMD, PCET	ROI-to-ROI	ACC, PCC	↑ Dynamic FC between ACC—PCC at 1w after Psi & baseline dynamic FC of the ACC was associated with less improvement in cognitive flexibility
Daws et al. [30] (1)º						
1st: 10 mg 2nd: 25 mg—Oral	None	Pre versus Post (+ 24 h)	BDI	NetC	N/A	↓Network modularity 24 h after Psi correlated with improvements in depression severity at 6 m ↓FC within DMN ↑FC between DMN & ECN (= FPN), SN
Daws et al. [30] (2)^‡^						
25 mg—Oral	Low dose of Psi (1 mg); daily placebo pills (microcrystalline cellulose)	Pre versus Post (+ 3w)	BDI	NetC, Other	N/A	↓Network modularity 3w after Psi correlated with improvements in depression severity; no changes in network modularity from baseline in escitalopram group ↑Dynamic flexibility in ECN (= FPN) correlated with improvements in depression symptoms at 3 weeks after Psi, but not in escitalopram group
Copa et al. [86]^‡^						
1st: 10 mg 2nd: 25 mg—Oral	None	Pre	QIDS	NetC	N/A	Baseline VisNet rsFC predicted early treatment response (+ 1-5w) Baseline DMN and ECN (= FPN) also predicted early treatment response (+ 2w) Baseline SN predicted sustained treatment response (+ 24w)

↑, increase; ↓, decrease; +, positive; -, negative; º, within-subjects design; *, crossover design; ^‡^, between-subjects design; H, hours; D, days; W, weeks; m, months; FC, functional connectivity; PPL, Plasma psilocin level; RSN, resting state network; ROIs, Regions of interest; SDI, Subjective Drug Intensity; VAS, Visual analogue scale; PANAS, Positive and negative affect schedule; STAI, State-trait anxiety inventory; QIDS, Quick inventory of depressive symptoms; BDI, Beck depression inventory; 5D-ASC, 5-Dimensional altered states of consciousness; 11D-ASC, 11-Dimensional altered states of consciousness; PEQ, Persisting effects questionnaire; GEMS-3, Geneve emotional music scale; DASS, Depression, Anxiety and Stress Scale; POMS, Profile of mood states; DPES, Dispositional positive emotion scale; MEQ, Mystical experience questionnaire; MEQ30, Revised mystical experience questionnaire; EDI, Ego dissolution inventory; SWSL, Satisfaction with life Scale; GRID-HAMD, GRID-Hamilton depression rating scale; PCET, Penn conditional exclusion test; NEO-PI-R, NEO Personality inventory—revised; MAAS, Mindfulness attention and awareness scale; MTL, Medial temporal lobe; ACC, Anterior cingulate cortex; PFC, Prefrontal cortex; vmPFC, ventromedial PFC; PCC, Posterior cingulate cortex; PHG, Parahippocampal gyrus; rSTG, Right superior temporal gyrus; lPCG, Left postcentral gyrus; IPC, Inferior parietal cortex; SBC, Seed-based connectivity; NetC, Network connectivity; DynC, Dynamic connectivity WBC, Whole-brain connectivity; ALFF, Amplitude of low-frequency fluctuations; DMN, Default mode network; TPN, Task positive network; VisN, Visual network; AudN, Auditory network; SMN, Sensorimotor network; DAN, Dorsal attention network; SN, Salience network; ECN, Executive control network; FPN, Fronto-parietal network

**Table 4:** Summary of task-based functional connectivity FC findings in psilocybin fMRI studies during cognitive/affective tasks. Includes studies involving healthy controls (n = 7) and depression patients (n = 4), with task paradigms, analyzed brain networks/regions, and a summary of results

Psi dose/admin	Placebo	Effects/scan timepoints	Task	Questionnaires	Data analysis	ROIs	Summary of findings
*Healthy participants*							
Carhart-Harris et al. [39]^○^							
2 mg—IV	Saline	Single acute	Memory retrieval	Unstandardized scales for memory vividness, emotional intensity, valence and visual imagery	WBC	N/A	↑ BOLD signal in Vis, AUD, SS, and parietal cortex during late phase memory processing
Kraehenmann et al. [41]º*							
0.16 mg/kg—Oral	Lactose	Single acute (+ 70–90 m)	Amygdala reactivity (fear discrimination) + simple motor task	PANAS, STAI, ASCq	SBC	AMG, Motor Cortex	↓ AMG response to positive and negative stimuli correlated with positive changes in mood
Kraehenmann et al. [41]º*							
0.16 mg/kg—Oral	Lactose	Single acute (+ 70–90 m)	Amygdala reactivity (fear discrimination) + simple motor task	PANAS, STAI	ROI-to-ROI	AMG, PFC, Visual Cortex	↓ V1 activity under Psi effects ↓ FC AMG – V1 during threat processing
Preller et al. [31]º*							
0.215 mg/kg—Oral	Maltose	Single acute (+ 75 m)	Social exclusion	5D-ASC, PANAS	WBC	N/A	↓ BOLD signal in MFG in response to social exclusion under Psi ↓ BOLD in dACC correlated with feeling of unity during social exclusion situations
Grimm et al. [53]º*							
0.16 mg/kg—Oral	Lactose	Single acute (+ 70–90 m)	Face discrimination	PANAS, STAI, ASCq	SBC	AMG	↓ FC right AMG – left Striatum for angry versus neutral faces ↓ FC right AMG – Frontal pole for happy versus neutral faces correlated with anxiety and depressive scores
Barrett et al. [44]º							
0.357 mg/kg—Oral	None	Pre (−1d) versus Post (+ 1w & 1 m)	Emotional discrimination, emotional recognition, emotional conflict (Stroop)	POMS, STAI, PANAS-X, DASS, DPES, BFI, TAS	WBC, ROI-to-ROI	AMG, ACC	↓ AMG BOLD signal 1w after Psi. Return to baseline 1 m later ↑ BOLD signal in the left ACC at 1w and increasing signal up to 1 m later ↑ BOLD signal in SS and fusiform gyrus 1 m after Psi ↑ global FC at 1w and 1 m post-Psi
Duerler et al. [80]º*							
0.2 mg/kg—Oral	Mannitol and colloidal silicon dioxide	Single acute (+ 85 m)	Somatosensory oddball	5D-ASC	Other	N/A	↓ BOLD signal vmPFC, dmPFC, V1 and Cerebellum in response to surprising stimuli
*TRMD/MDD patient*s							
Roseman et al. [29]º							
1st: 10 mg 2nd: 25 mg—Oral	None	Pre (−1w) versus Post (+ 24 h)	Emotional discrimination	In-scanner “state” ratings, BDI, QIDS, STAI,	SBC	AMG	↑ right AMG reactivity to fearful & happy versus neutral faces post-Psi ↑ right AMG reactivity to fearful versus neutral faces predicted clinical improvements at 1w post-Psi
Mertens et al. [74]º							
1st: 10 mg 2nd: 25 mg—Oral	None	Pre (−1w) versus Post (+ 24 h)	Emotional discrimination	BDI, QIDS, RRS, STAI	SBC	AMG, PFC	↓ FC between vmPFC & right AMG correlated with ↓ rumination at 1w post-Psi
Shukuroglou et al. [105]º							
10 mg/25 mg—Oral	None	Pre (−1w) versus Post (+ 24 h)	Music listening	SHAPS, GEMS	SBC	NAcc	↓ FC between NAcc and DMN areas during music (vs. no music) listening 24 h after Psi
Wall et al. [77]º							
10 mg/25 mg—Oral	None	Pre (−1w) versus Post (+ 24 h)	Music listening	5D-ASC, GEMS	ALFF, ROI-to-ROI	LG, STG	↑ ALFF during music listening after Psi in the STG, positively correlated with various sub-scores of the 5D-ASC ↓ ALFF during rest after Psi in the medial frontal lobe

↑, increase; ↓, decrease; º, within-subjects design; *, crossover design; ‡, between-subjects design; H, hours; D, days; W, weeks; M, months; FC, functional connectivity; ROIs, Regions of interest; VAS, Visual analogue scale; PANAS, Positive and negative affect schedule; PANAS-X, Positive and negative affect schedule, Form X; STAI, State-trait anxiety inventory; ASCq, altered states of consciousness questionnaire; QIDS, Quick inventory of depressive symptoms, BDI, Beck depression inventory; 5D-ASC, 5-Dimensional altered states of consciousness; SHAPS, Snaith-hamilton pleasure scale; GEMS, Geneva emotional music scale; POMS, Profile of mood states; DASS, Depression, Anxiety, and Stress Scale; DPES, Dispositional Positive Emotion Scale; BFI, Big Five Inventory; TAS, Tellegen Absorption Scale; RRS, Ruminative Response Scale; SBC, Seed-based connectivity; NetC, network connectivity; DynC, Dynamic connectivity; WBC, Whole-brain connectivity; ALFF, Amplitude of low-frequency fluctuations; AMG, Amygdala; ACC, Anterior cingulate cortex; dACC, dorsal ACC; MFG, middle frontal gyrus; PFC, Prefrontal cortex; dmPFC, dorsomedial PFC; vmPFC, ventromedial PFC; PCC, Posterior cingulate cortex; PH, Parahippocampus; Vis, Visual cortex; V1, Primary visual cortex; AUD, Auditory cortex; SS, Somatosensory cortex; NAcc, Nucleus accumbens; LG, Lingual gyrus; STG, Superior temporal gyrus, SMG, Supramarginal gyrus

Of the 20 original datasets, 13 incorporated a placebo-controlled design, although the type of placebo varied among studies (see Tables 3 and 4). Some studies adopted a potentially controversial design in which placebo and Psi administrations occurred on the same day, only 4 h apart [25, 26]. Unbeknownst to participants, the first administration was always the placebo (a very low and non-psychoactive dose of Psi) followed by a low-moderate dose of Psi.

Doss, Povazan [18] (2) compared the FC effects of Psi with those of the conventional antidepressant escitalopram, and blinded participants by administering a low dose of Psi to the escitalopram group and daily placebo pills to the Psi group to mimic the daily administration of escitalopram.

### Statistical approach

The main difference between the studies encompassed in this review lies in their methodologies. Considerable variability is evident in the statistical approaches employed by each study (see Table 3 and 4). The majority of studies employed seed-based connectivity (n = 8) or ROI-to-ROI analysis (n = 6), both of which rely on predefined ROIs. Additionally, several studies employed traditional approaches such as network connectivity (n = 11) and whole-brain connectivity (n = 6).

#### Regions of interest

Across studies, the amygdala emerged as the most frequently used ROI, both in research involving patients with depression and studies with healthy participants (Table 3 and 4). The anterior cingulate cortex (ACC) and the PFC were the second most frequently studied regions. Additionally, the thalamus, the posterior cingulate cortex (PCC) and the parahippocampus (PH) are used as ROIs in more than one study.

Among the studies focusing on the amygdala, 6 of 7 employed task-based FC designs, all centered on emotion-related tasks. Only one study analyzing resting-state FC (rsFC) used the Amygdala as a ROI [28]. Results are detailed below in Section 4.2.1.

#### Within and between–network FC

10 studies focused on analyzing the FC within and between predefined brain functional networks. This approach typically involves assessing the different connectivity produced between brain regions (hubs) associated with specific functional networks under two conditions—in this case placebo and Psi. Among the studies evaluated, all analyzed resting-state networks (RSNs), the most common being the default mode network (DMN) and the frontoparietal network (FPN) (or executive control network, ECN) (see Table 3).

#### Other analyses of FC

Among the 5 studies that employed alternative methods to analyze FC, the methodology varied. Lebedev, Lovden [32] grouped 200 ROIs into 5 distinct “communities” and reported a robust negative correlation between the diversity of functional connections in the anterior PH cortex and the intensity of ego-dissolution experiences.

Another study aimed to characterize the dynamics of blood oxygenation-level-dependent signal (BOLD) phase coherence using an innovative approach, comparing BOLD phase-locking patterns with canonical resting-state functional networks. Their results indicated an association between the FPN/ECN and the psychoactive effects induced by Psi, supporting the notion that Psi induces an overly integrated and highly stable brain state [33].

Another study investigated Psi-dependent changes in cortical gradients and functional hierarchy using gradient-mapping approaches. Cortical regions were classified as unimodal or transmodal cortices, representing association or sensorimotor regions, respectively. The results indicated a shift in both cortex types toward greater harmonization of their FC patterns [34].

The dynamics of FC during Psi acute effects were correlated with plasma psilocin levels in a recent study. A negative correlation was identified between both blood psilocin levels and subjective drug intensity scores with the fractional occurrence of the FPN/ECN. Furthermore, reduced FPN rsFC with the cingulum, the ventromedial PFC (vmPFC), and parieto-occipital fissure were reported, slightly overlapping regions typically associated with the DMN [35].

## Discussion

To our knowledge, this is the first review to systematically examine the effects of Psi on large-scale brain networks. Converging evidence from neuroimaging studies indicates that Psi induces widespread alterations in FC, promoting a more integrated and flexible brain state. These effects are observed across key resting-state networks, including the DMN, salience network (SN), ECN (or FPN), and visual network, with potential implications for both acute and sustained therapeutic outcomes. Despite a significant increase in Psi research over the last decade, functional neuroimaging studies remain relatively sparse. To date, slightly more than 350 subjects have been assessed across only 36 original research articles. These figures underscore the need for further research to establish consistency and validity in the reported findings.

### Methodological heterogeneity

The reviewed studies exhibit substantial variability in study design, including differences in dose, route of administration, scan timing, analytic methods, and participant characteristics. This heterogeneity is particularly notable given the limited number of studies providing new datasets, and it critically limits the ability to compare results across studies or draw definitive conclusions. Despite this variability, several findings appear robust and consistent across methodological differences. In healthy participants, decreased amygdala reactivity to negative stimuli and reduced mPFC functional connectivity, observed in both resting-state and task-based designs, consistently emerge across studies (see Sect. 4.2). Alterations in large-scale networks, such as DMN mPFC-PCC decoupling and reduced modularity, as well as increased between-network connectivity between the ECN/FPN and other resting state networks have also been repeatedly observed (see Sect. 4.3). These effects appear robust across healthy and clinical participants, resting-state and task-based paradigms, different psychedelics and doses, scan timings, and analytic approaches.

In contrast, other effects, such as ACC connectivity patterns, thalamocortical connectivity, within-network ECN/FPN changes, and amygdala reactivity in TRMD patients, appear highly sensitive to study design, including participant population, task versus resting-state conditions, and analytic techniques. These results underscore the need for replication studies with standardized methodologies to delineate robust neurobiological effects of Psi from design-dependent variability.

Of note, participant age may be an important factor to address in future research. It would be valuable to examine younger and older populations separately to assess whether the therapeutic effects are comparable in the developing brains of individuals under 25 years old and in the aging brains of those over 65 years old [36–38]. Such analyses could help identify specific age ranges in which Psi-assisted therapy is safest and most effective.

Another key factor to consider is the participants’ history of psychedelic use. Some studies in this review provided details regarding lifetime use frequency and time since last use, whereas six studies did not specify this information at all. Studies with a more homogeneous sample of participants (with or without previous psychedelic experience) reduce potential confounding factors, making their results more robust. Similarly, the inclusion of more details about prior pharmacological interventions could enhance our understanding of which MDD/TRMD patients would benefit most from Psi-assisted therapy.

Only nine studies included cognitive tasks into their designs. This is understandable, as many represented the initial acquisitions of Psi effects using fMRI, when clear hypotheses about its impact on brain connectivity were still limited. However, when evaluating the potential benefits of Psi-assisted psychotherapy in psychiatric disorders, integrating cognitive performance measures with FC data, both before and after treatment, is essential for establishing a neurobiological foundation for the clinical improvements frequently reported in Psi-assisted psychotherapy.

Notably, there is considerable variability in the timing of Psi administration and fMRI acquisition across studies (see Table 3 and 4), with these parameters often insufficiently explained or justified. Only one study administered Psi intravenously [39], and the resulting dataset has since become the most frequently reused. In that case, the effects were predicted to begin instantaneously, enabling analysis of their onset [40]. In contrast, studies investigating the acute effects of orally administered Psi differed in the time window between administration and scanning. Some studies based their timing on previous research, initiating scans between 70 and 90 min post-Psi administration [41], while others began at 40 min [42] or 100 min [25] after administration. One study adopted a longer time window, scanning at 40, 80, 130 and 300 min after Psi administration [43], allowing for a more precise characterization of the time course of FC changes.

Although limited in number, studies including follow-up scans suggest that Psi-induced effects on the brain can persist well beyond the elimination of the drug from the body, lasting up to 4 weeks after intake [44, 45]. Moreover, significant changes in questionnaire scores have been reported up to 3 months after Psi administration [46]. Additional studies incorporating follow-up scans would help clarify whether short-term effects reflect enduring neurobiological changes and how they relate to clinical outcomes. Such evidence is essential for determining the optimal dosing frequency in Psi-assisted therapies, and would mark a meaningful advancement over current pharmacological treatments that require chronic daily ingestion.

There has been speculation that psychedelic trials may overestimate treatment effects due to expectancy and unblinding issues [47, 48]. To minimize these biases, careful selection of appropriate placebos is essential. Active placebos, such as low doses of psychedelics or other psychoactive compounds that produce perceptible but comparable psychoactive effects, are considered more effective in reducing expectancy-related bias than inert placebos [49]. In the present review, only 3 studies used an active placebo [25, 26, 30], thereby enhancing the robustness of their findings by mitigating expectancy effects.

None of the included studies reported pre-study statistical power calculations to justify their sample sizes, limiting the reliability and generalizability of their conclusions. Given the typically small cohorts, incorporating such calculations would enhance the statistical power and strengthen result validity [50]. Only 5 articles included data from more than 25 participants [26, 27, 41, 42, 51], offering comparatively more robust data than the average fMRI study [50]. Nevertheless, no articles were excluded or discounted in this review due to the proven reliability of neuroimaging methods, particularly fMRI [52]. However, future research should prioritize larger sample sizes and, whenever possible, employ multi-center designs to strengthen the statistical validity and reproducibility of the findings.

### Effects of Psi on ROIs

#### Amygdala reactivity

All studies in this review involving healthy participants reported decreased amygdala reactivity to negative emotional stimuli, as well as reduced FC between the amygdala and key regions involved in salience and emotional valence processing under Psi effects [44, 51, 53]. These findings are consistent with those observed in LSD [54] and ayahuasca [55], and may be attributable to the 5-HT_2A_R agonism these substances exert in multiple subregions of the amygdala [54, 56]. In addition, administration of selective serotonin reuptake inhibitors (SSRIs) to healthy subjects has similarly been shown to reduce amygdala reactivity to negatively valenced stimuli [57], possibly reflecting the indirect serotonergic agonism of SSRIs within the amygdala.

Nevertheless, among patients with TRMD, one study in this review reported increased amygdala reactivity to fearful versus neutral faces following Psi administration [29], suggesting that Psi may differentially affect amygdala reactivity in this population. What remains to be investigated is whether these effects, if replicated, are ultimately beneficial to patients. Considerable evidence indicates that SSRIs normalize amygdala hyperreactivity to negative stimuli and amygdala hypoactivity to positive stimuli in depression [58–60]. Similarly, ketamine administration in TMRD patients has been shown to reduce amygdala reactivity to negatively valenced stimuli [61]. Another study included in this review found no significant alterations in amygdala rsFC 24 h after Psi treatment in TRMD patients [28], a less surprising result given that changes in amygdala reactivity appear to depend on the emotional valence of the stimuli and may not be detectable under task-free conditions [60]. Further investigation on amygdala reactivity during and after Psi effects in depression is needed to elucidate the described discrepancies.

#### Anterior cingulate cortex

The ACC is critically involved in emotion and salience processing, anxiety, social pain, negative rumination and depression [44, 62–66]. Among the four studies in this review that examined ACC FC in healthy participants, findings were mixed (see Tables 3 and 4). Similar inconsistencies have been reported in ayahuasca studies [67, 68], suggesting that ACC FC may be particularly sensitive to variations in experimental methodologies and participant characteristics.

One study in this review observed increased connectivity between the ACC and hippocampus in healthy participants during Psi effects [40]. This data aligns with prior research on ayahuasca, which reported enhanced coupling between the ACC, hippocampus, parahippocampus, and amygdala in pre- versus post-treatment comparisons [69]. While further investigation is needed to determine whether this increased coupling persists during the “after-glow” of Psi, it is possible that a key antidepressant effect of Psi involves the enhanced interplay between the ACC, which integrates cognitive and emotional information, and limbic structures involved in emotional and memory processing.

Two studies in this review reported increased coupling between the ACC and PCC in patients with TRMD and MDD, respectively [18, 28]. Given that prior research has shown reduced ACC-PCC connectivity in MDD patients at baseline compared to healthy controls [70], these findings suggest that Psi may help normalize FC between these regions.

#### Prefrontal cortex

All studies in this review involving healthy participants found decreased FC in the medial PFC (mPFC), both in resting state conditions and during a tactile mismatch processing task under Psi effects. These findings align with previous research showing that LSD and ayahuasca induce hypo-connectivity in the mPFC and other associative cortical areas [67, 71], supporting the notion that serotonergic psychedelics provoke an acute desynchronization across associative networks.

Two studies further reported reduced rsFC between the mPFC and the PCC, observed acutely in the general population and 2 days post-Psi in expert meditators [27, 39]. The latter study also found an association between mPFC-PCC decoupling and positive states of ego dissolution, consistent with findings in experienced meditators administered LSD intravenously [72]. As key hubs of the DMN, the mPFC and PCC play a critical role in self-referential processing, suggesting that Psi-induced alterations in their FC may underlie changes in self-perception [27]. The mPFC is also among the brain regions exhibiting the greatest inter-subject heterogeneity in FC patterns under Psi, alongside the PCC and Inferior Parietal Cortex (IPC). This suggests that Psi reconfigures brain connectivity in an individual-specific manner, shifting the balance from frontoparietal to DMN involvement. These altered connectivity patterns may predict the subjective intensity of the psychedelic experience [73].

In patients with TRMD, Psi reduced FC between the mPFC and right amygdala during an emotion discrimination task that was correlated with decreased rumination at 1 week [74]. Additionally, reduced rsFC between the parahippocampal gyrus (PHG) and PFC predicted a reduction in depression scores 5 weeks post-treatment [28]. Patients with depression typically show elevated PHG-PFC rsFC [75] and impaired prefrontal-amygdala inhibitory circuitry during affective reappraisal [76] suggesting that Psi may help normalize PFC-limbic circuitry. Psi administration also decreased low-amplitude frequency fluctuations in the medial frontal areas during resting state, indicating a direct regional effect. Moreover, the same study demonstrated that Psi treatment alters patients' perception of music by directly modulating auditory processing, offering new insights into Psi’s bottom-up effects on sensory integration [77].

#### Thalamus

Despite its crucial role in filtering and distributing information between subcortical and cortical areas [78], only 3 studies in this review evaluated the effects of Psi on the thalamus, all conducted in healthy participants during resting state (see Table 3). Two studies reported contradictory results in thalamocortical connectivity [26, 79]; these discrepancies may be attributable to differences in analytical methodology (whole-thalamus vs. Independent Component Analysis, respectively) and the inclusion of participants with long-term meditation practices in the latter study, which could have influenced FC more than initially anticipated. Another study observed a decrease in intrathalamic FC and cerebral blood flow [39], suggesting that Psi may reduce thalamic functionality and contribute to altered thalamocortical connectivity. Consistent with the CSTC model, Psi appears to modulate CSTC circuitry, leading to thalamic disinhibition and distorting somatosensory information processing without affecting cortical-level somatosensory perception [80]. The thalamus has been described to be anatomically and functionally altered in MDD patients, with notable impairments in connectivity to key frontal cortex regions, including the orbitofrontal cortex [81, 82], as well as increased rsFC with the cingulate and the precuneus [83]. Consequently, these findings underscore the need for further investigation in both healthy and clinical populations to fully comprehend the complex effects of Psi on thalamic and thalamocortical FC.

### Effects of Psi on network connectivity

In network connectivity analyses, the DMN emerges as the most studied network. Multiple studies in this review reported decreases in rsFC within the DMN in both healthy participants [34, 42, 45, 73] and patients with TRMD [30] (1) under Psi. These findings align with previous literature showing similar effects in healthy participants that were administered ayahuasca [67] and LSD [71, 72], as well as TRMD patients who were administered ketamine [61]. In patients with MDD, past findings suggest that the DMN typically exhibits hyperconnectivity and increased modularity during resting state conditions, reflecting maladaptive rumination [75, 84]. Notably, one study in our sample found an increase in within-DMN FC in patients with TRMD 24 h post-Psi [28]. Furthermore, the DMN appears to show increased coupling with other canonical resting-state networks in healthy participants under Psi [43, 85] and in TRMD patients following Psi [30] (2), consistent with prior findings in ayahuasca [68] and LSD [71, 72]. Similar effects have been reported with ketamine [61] suggesting that psychedelics may enable the brain to access a wider dynamic repertoire of metastable sub-states [17]. Collectively, these findings support the REBUS model, indicating that Psi disrupts the DMN during resting state, decreases network modularity, and enhances integration between typically segregated brain networks. In depression, such effects may help reduce pathological DMN activity through promoting a more flexible and desynchronized brain state.

Interestingly, one study in our sample reported reduced FC within the claustrum, as well as between the right claustrum and DMN. Moreover, DMN integrity was correlated with the connectivity of the right claustrum to the DMN [25]. This finding aligns with the CCC model, suggesting that Psi can disrupt high-level cortical networks via claustro-cortical circuits, potentially contributing to the subjective effects of psychedelic experiences [18].

The FPN/ECN appears to undergo rapid desynchronization during acute Psi effects, resulting in decreased top-down control systems and increased distractibility in participants. Two studies reported reduced within-FPN connectivity during Psi administration [33, 35], while two others observed increased rsFC between the DMN and FPN [42, 85]. Roseman, Leech [85] also found enhanced rsFC between the FPN and the visual network, the auditory network, the sensorimotor network (SMN), and the ECN during acute Psi effects.

In patients with depression, reduced rsFC and task-based FC in the FPN have been reported [75, 84]. Although Psi reduces within-FPN FC, its effects on this network in MDD patients have not yet been investigated. Given Psi’s general impact on all networks—reducing modularity while increasing between-network integration—the reduction of within-FPN FC may represent a necessary adjustment for general network synchronization. Nevertheless, further investigation is required to replicate these findings and clarify their functional significance.

Two studies in our sample suggest that Psi induces an acute increase in within-ECN/FPN FC followed by a subsequent decrease at 1 week post-Psi and potentially even longer [43, 46]. Regarding between-network connectivity, all studies indicate that Psi increases FC between the ECN/FPN and other canonical resting state networks including the DMN, DAN, and SMN, in both healthy participants and TRMD patients [30, 43, 85]. These findings are consistent with evidence suggesting that the psychedelic state reduces network segregation and promotes a more globally integrated brain state [34, 42, 54, 71, 79]. Additionally, baseline ECN and DMN rsFC have been shown to predict early treatment response at two weeks in TRMD patients [86].

Although the SN has been less extensively studied under Psi, some reports have identified notable changes [75, 87]. Particularly, enhanced rsFC between the DMN and the SN have been observed in both MDD patients and healthy participants [30, 42], while decreased modularity within the SN has been reported during Psi effects [32]. Moreover, decreased rsFC between the insula, the PFC and the temporoparietal junction has also been documented [88]. Given that the Insula is a primary hub of the SN and plays a critical role in addiction [89], increased rsFC may reflect a compensatory mechanism for deficits observed in addiction disorders, consistent with reports of attenuated cerebral blood flow in the insula associated with SN impairment in alcohol use disorder [90].

Furthermore, the ACC, a key region in both acute and sustained Psi effects [18, 28, 40, 44], is another essential hub of the SN [91, 92]. Recent findings by Copa, Erritzoe [86] indicate that baseline rsFC in the SN correlates with sustained treatment response up to 24 weeks post-treatment in TRMD patients. Collectively, the neuroimaging results from the studies in this review, alongside findings reported by Bogenschutz, Forcehimes [3], Garcia-Romeu, Griffiths [93], Dos Santos, Bouso [20], and Bogenschutz, Ross [94], highlight the potential value of continued research into Psi-assisted psychotherapy for the treatment of addictive disorders.

Finally, another study investigated the visual network to examine how Psi affects sensory input and associative processes. The study reported an increase in within-network inhibition in both primary visual and visual-association regions. Coupled with enhanced top-down inhibitory connectivity and reduced bottom-up connectivity, these findings suggest that Psi reduces the influence of external stimuli while amplifying top-down inferences, resembling the process of dreaming [95]. Furthermore, pre-treatment visual network rsFC has been proposed as a predictor of early treatment response, occurring between one and five weeks, in patients with TRMD [86].

### Spatial parcellation of networks

The studies examining Psi’s effects on network connectivity defined the RSNs using either ICA or atlases. Among the 12 studies that employed network atlases, 7 different atlases were referenced [96–102]. These atlases varied in the number of networks described (ranging from 7–13), as well as in the labels and descriptions of the voxels making up each network. Notably, there is a significant overlap between the voxels used to delineate the ECN and FPN, with the majority of atlases referring to one network or the other [96, 97, 101], and one atlas including both [99]. Two atlases do not include a SN [99, 101], while two others distinguished the dorsal attention from the ventral attention network [96, 101]. The DMN is included in all atlases; however, Roseman, Leech [85] incorporated another ICA component from the Smith, Fox [99] atlas, labelling it DMN2 (an anterior DMN and ECN hybrid). This inconsistency in network nomenclature, compounded by overlapping ROIs between networks, complicates direct comparisons and interpretation of FC changes among RSNs [61].

### Psi in depression treatment

One of the primary objectives of investigating Psi effects is its potential role as a catalyst in psychotherapy for MDD [8, 10, 20]. Despite growing interest, there is a notable scarcity of studies providing long-term fMRI data. Among the studies included in this review that assessed patients with depression, none incorporated follow-up fMRI scans; instead, they relied solely on self-report questionnaires collected from 1 week up to 6 months post-treatment [18, 28–30]. Including longitudinal imaging alongside behavioral measures would help determine whether sustained improvements in clinical outcomes are mirrored by changes in FC.

In resting-state settings, Psi-assisted therapy for MDD has been associated with increased vmPFC-IPC rsFC, which predicted treatment response at 5 weeks but did not correlate with clinical questionnaire scores [18, 28]. Doss, Povazan [18] revealed that baseline dynamic FC predicted improved cognitive flexibility at 1 and 4 weeks. Although these effects were not clinically significant, they support the concept of increased neuroplasticity following psychedelic use [15] and suggest that ongoing integration psychotherapy may be particularly beneficial during this window.

Decreases in network modularity one day after Psi-assisted psychotherapy were predictive of substantial reductions in depression severity at 6 months [30]. The same study reported similar results from another trial, where decreased network modularity at 3 weeks post-treatment correlated with improvements in depressive symptoms, outperforming standard medication. Despite some variations in network recruitment patterns, these results align with numerous studies in healthy participants investigating acute Psi effects on network integrity [25, 32, 43]. While classic antidepressants have also been shown to reduce the rsFC of the DMN in MDD patients, they generally produce limited changes in between-network connectivity [103, 104]. Collectively, these results suggest that Psi’s distinct effect lies in its generalized reduction of overall network modularity, promoting greater network integration.

## Conclusions

After reviewing all available fMRI studies, it is evident that there is a substantial lack of methodological coherence across the literature. The analyzed studies employ highly heterogeneous designs—differing in dose, route of administration, scan timing, analytical approaches, and participant characteristics—making cross-study comparisons and generalization of findings difficult. To enhance the reproducibility and interpretability of future research, we propose the following methodological recommendations:

- Inclusion of larger sample sizes and prior power calculations to ensure statistical robustness and reduce false-positive findings.
- Incorporation of cognitive tasks, particularly when evaluating psychiatric populations, to better link neuroimaging outcomes with behavioral and clinical effects.
- Inclusion of follow-up scanning sessions at multiple timepoints to assess the temporal stability of Psi-induced brain changes.
- Employment of active placebos to reduce expectancy and unblinding effects.
- Inclusion of adequate wash-out periods of at least 4 weeks in crossover designs to prevent carry-over effects.
- Detailed participant profiles, including prior psychedelic use, past pharmacological interventions (if relevant), and relevant clinical background, to improve the interpretability and reproducibility of findings.

Head motion during scanning sessions was commonly not addressed in the reviewed articles. Variations in quality controls and preprocessing, especially motion-correction strategies, may influence the results and conclusions of each study. We recommend that future studies explicitly report and justify their preprocessing steps in the methods section.

Currently, our comprehension of brain FC dynamics and their modulation under Psi remains limited. Greater methodological alignment across studies is needed; in particular, establishing standardized timing between Psi administration and fMRI acquisition would facilitate both comparability and replication when assessing acute Psi effects. Furthermore, most studies do not describe or differentiate the type of psychological support or psychotherapy model employed, making it difficult to disentangle the specific contributions of Psi from those of the therapeutic context.

Future studies should aim to include larger sample sizes to increase statistical power and reliability of results, while also minimizing dropouts due to head motion in the scanner. Additionally, the inclusion of appropriate control or placebo groups is equally important to distinguish the effects of psychotherapy alone and to mitigate expectancy effects. Finally, the incorporation of longitudinal follow-up scans is critically relevant. This represents a major gap in the current literature, as objective neurobiological measures are essential to support complement and validate clinical questionnaires, which alone may provide an incomplete assessment of treatment efficacy**.**

In depression treatment, an interval of approximately 1 month between Psi-administered sessions could be a suitable approach, as several studies have reported sustained clinical improvements up to 3 months post-administration, even when FC changes are no longer detectable. Nevertheless, further research is required to define the optimal dosing and timing protocols, supported by neurobiological evidence rather than relying solely on clinical scores.

## Data Availability

No datasets were generated or analysed during the current study.

## References

[1] Nichols DE. Psychedelics. Pharmacol Rev. 2016;68(2):264–355. 26841800 10.1124/pr.115.011478 PMC4813425

[2] Guzmán G. Species diversity of the genus Psilocybe (Basidiomycotina, Agaricales, Strophariaceae) in the world mycobiota, with special attention to hallucinogenic properties. Int J Med Mushrooms. 2005;7(1 & 2):305–32.

[3] Bogenschutz MP, et al. Psilocybin-assisted treatment for alcohol dependence: a proof-of-concept study. J Psychopharmacol. 2015;29(3):289–99. 25586396 10.1177/0269881114565144

[4] Lowe H, et al. The therapeutic potential of psilocybin. Molecules. 2021;26(10):2948. 34063505 10.3390/molecules26102948 PMC8156539

[5] Spriggs MJ, et al. Study protocol for “Psilocybin as a treatment for anorexia nervosa: a pilot study.” Front Psych. 2021;12:735523. 10.3389/fpsyt.2021.735523 PMC8563607 34744825

[6] Carhart-Harris R, et al. Trial of psilocybin versus escitalopram for depression. N Engl J Med. 2021;384(15):1402–11. 33852780 10.1056/NEJMoa2032994

[7] Gukasyan N, et al. Efficacy and safety of psilocybin-assisted treatment for major depressive disorder: prospective 12-month follow-up. J Psychopharmacol. 2022;36(2):151–8. 35166158 10.1177/02698811211073759 PMC8864328

[8] Goodwin GM, et al. Single-dose psilocybin for a treatment-resistant episode of major depression. N Engl J Med. 2022;387(18):1637–48. 36322843 10.1056/NEJMoa2206443

[9] Passie T, et al. The pharmacology of psilocybin. Addict Biol. 2002;7(4):357–64. 14578010 10.1080/1355621021000005937

[10] Ling S, et al. Molecular mechanisms of psilocybin and implications for the treatment of depression. CNS Drugs. 2022;36(1):17–30. 34791625 10.1007/s40263-021-00877-y

[11] Jaster AM, Gonzalez-Maeso J. Mechanisms and molecular targets surrounding the potential therapeutic effects of psychedelics. Mol Psychiatry. 2023;28(9):3595–612. 37759040 10.1038/s41380-023-02274-x PMC11078317

[12] Madsen MK, et al. Psychedelic effects of psilocybin correlate with serotonin 2A receptor occupancy and plasma psilocin levels. Neuropsychopharmacol. 2019;44(7):1328–34. 10.1038/s41386-019-0324-9 PMC6785028 30685771

[13] Carter OL, et al. Modulating the rate and rhythmicity of perceptual rivalry alternations with the mixed 5-HT2A and 5-HT1A agonist psilocybin. Neuropsychopharmacol. 2005;30(6):1154–62. 10.1038/sj.npp.1300621 15688092

[14] Pokorny T, et al. Modulatory effect of the 5-HT1A agonist buspirone and the mixed non-hallucinogenic 5-HT1A/2A agonist ergotamine on psilocybin-induced psychedelic experience. Eur Neuropsychopharmacol. 2016;26(4):756–66. 26875114 10.1016/j.euroneuro.2016.01.005

[15] Vollenweider FX, Preller KH. Psychedelic drugs: neurobiology and potential for treatment of psychiatric disorders. Nat Rev Neurosci. 2020;21(11):611–24. 32929261 10.1038/s41583-020-0367-2

[16] Carhart-Harris RL, Friston KJ. REBUS and the anarchic brain: toward a unified model of the brain action of psychedelics. Pharmacol Rev. 2019;71(3):316–44. 31221820 10.1124/pr.118.017160 PMC6588209

[17] Gattuso JJ, et al. Default mode network modulation by psychedelics: A systematic review. Int J Neuropsychopharmacol. 2023;26(3):155–88. 36272145 10.1093/ijnp/pyac074 PMC10032309

[18] Doss MK, et al. Psilocybin therapy increases cognitive and neural flexibility in patients with major depressive disorder. Transl Psychiatry. 2021;11(1):574. 34750350 10.1038/s41398-021-01706-y PMC8575795

[19] Reiff CM, et al. Psychedelics and psychedelic-assisted psychotherapy. Am J Psychiatry. 2020;177(5):391–410. 32098487 10.1176/appi.ajp.2019.19010035

[20] Dos Santos RG, et al. Efficacy, tolerability, and safety of serotonergic psychedelics for the management of mood, anxiety, and substance-use disorders: a systematic review of systematic reviews. Expert Rev Clin Pharmacol. 2018;11(9):889–902. 30102078 10.1080/17512433.2018.1511424

[21] McCulloch DE, et al. Psychedelic resting-state neuroimaging: A review and perspective on balancing replication and novel analyses. Neurosci Biobehav Rev. 2022;138:104689. 35588933 10.1016/j.neubiorev.2022.104689

[22] Dos Santos RG, et al. Classical hallucinogens and neuroimaging: A systematic review of human studies: Hallucinogens and neuroimaging. Neurosci Biobehav Rev. 2016;71:715–28. 27810345 10.1016/j.neubiorev.2016.10.026

[23] Liberati A, et al. The PRISMA statement for reporting systematic reviews and meta-analyses of studies that evaluate health care interventions: explanation and elaboration. PLoS Med. 2009;6(7):e1000100. 19621070 10.1371/journal.pmed.1000100 PMC2707010

[24] Radua J. PRISMA 2020 - An updated checklist for systematic reviews and meta-analyses. Neurosci Biobehav Rev. 2021;124:324–5. 33596413 10.1016/j.neubiorev.2021.02.016

[25] Barrett FS, et al. Psilocybin acutely alters the functional connectivity of the claustrum with brain networks that support perception, memory, and attention. Neuroimage. 2020;218:116980. 32454209 10.1016/j.neuroimage.2020.116980 PMC10792549

[26] Gaddis A, et al. Psilocybin induces spatially constrained alterations in thalamic functional organizaton and connectivity. Neuroimage. 2022;260:119434. 35792293 10.1016/j.neuroimage.2022.119434 PMC10749714

[27] Smigielski L, et al. Psilocybin-assisted mindfulness training modulates self-consciousness and brain default mode network connectivity with lasting effects. Neuroimage. 2019;196:207–15. 30965131 10.1016/j.neuroimage.2019.04.009

[28] Carhart-Harris RL, et al. Psilocybin for treatment-resistant depression: fMRI-measured brain mechanisms. Sci Rep. 2017;7(1):13187. 29030624 10.1038/s41598-017-13282-7 PMC5640601

[29] Roseman L, et al. Increased amygdala responses to emotional faces after psilocybin for treatment-resistant depression. Neuropharmacology. 2018;142:263–9. 29288686 10.1016/j.neuropharm.2017.12.041

[30] Daws RE, et al. Increased global integration in the brain after psilocybin therapy for depression. Nat Med. 2022;28(4):844–51. 35411074 10.1038/s41591-022-01744-z

[31] Preller KH, et al. Effects of serotonin 2A/1A receptor stimulation on social exclusion processing. Proc Natl Acad Sci U S A. 2016;113(18):5119–24. 27091970 10.1073/pnas.1524187113 PMC4983864

[32] Lebedev AV, et al. Finding the self by losing the self: Neural correlates of ego-dissolution under psilocybin. Hum Brain Mapp. 2015;36(8):3137–53. 26010878 10.1002/hbm.22833 PMC6869189

[33] Lord LD, et al. Dynamical exploration of the repertoire of brain networks at rest is modulated by psilocybin. Neuroimage. 2019;199:127–42. 31132450 10.1016/j.neuroimage.2019.05.060

[34] Girn M, et al. Serotonergic psychedelic drugs LSD and psilocybin reduce the hierarchical differentiation of unimodal and transmodal cortex. Neuroimage. 2022;256:119220. 35483649 10.1016/j.neuroimage.2022.119220

[35] Olsen AS, et al. Psilocybin modulation of time-varying functional connectivity is associated with plasma psilocin and subjective effects. Neuroimage. 2022;264:119716. 36341951 10.1016/j.neuroimage.2022.119716

[36] Dowling GJ, Weiss SR, Condon TP. Drugs of abuse and the aging brain. Neuropsychopharmacol. 2008;33(2):209–18. 10.1038/sj.npp.1301412 17406645

[37] Gogtay N, et al. Dynamic mapping of human cortical development during childhood through early adulthood. Proc Natl Acad Sci USA. 2004;101(21):8174–9. 15148381 10.1073/pnas.0402680101 PMC419576

[38] Rapoport JL, Gogtay N. Brain neuroplasticity in healthy, hyperactive and psychotic children: insights from neuroimaging. Neuropsychopharmacol. 2008;33(1):181–97. 10.1038/sj.npp.1301553 17851542

[39] Carhart-Harris RL, et al. Neural correlates of the psychedelic state as determined by fMRI studies with psilocybin. Proc Natl Acad Sci USA. 2012;109(6):2138–43. 22308440 10.1073/pnas.1119598109 PMC3277566

[40] Tagliazucchi E, et al. Enhanced repertoire of brain dynamical states during the psychedelic experience. Hum Brain Mapp. 2014;35(11):5442–56. 24989126 10.1002/hbm.22562 PMC6869695

[41] Kraehenmann R, et al. Psilocybin-induced decrease in amygdala reactivity correlates with enhanced positive mood in healthy volunteers. Biol Psychiatry. 2015;78(8):572–81. 24882567 10.1016/j.biopsych.2014.04.010

[42] Mason NL, et al. Spontaneous and deliberate creative cognition during and after psilocybin exposure. Transl Psychiatry. 2021;11(1):209. 33833225 10.1038/s41398-021-01335-5 PMC8032715

[43] Madsen MK, et al. Psilocybin-induced changes in brain network integrity and segregation correlate with plasma psilocin level and psychedelic experience. Eur Neuropsychopharmacol. 2021;50:121–32. 34246868 10.1016/j.euroneuro.2021.06.001

[44] Barrett FS, et al. Emotions and brain function are altered up to one month after a single high dose of psilocybin. Sci Rep. 2020;10(1):2214. 32042038 10.1038/s41598-020-59282-y PMC7010702

[45] Siegel JS, et al. Psilocybin desynchronizes the human brain. Nature. 2024;632(8023):131–8. 39020167 10.1038/s41586-024-07624-5 PMC11291293

[46] McCulloch DE, et al. Lasting effects of a single psilocybin dose on resting-state functional connectivity in healthy individuals. J Psychopharmacol. 2022;36(1):74–84. 34189985 10.1177/02698811211026454 PMC8801642

[47] Madras B. Psilocybin in treatment-resistant depression. N Engl J Med. 2022;387(18):1708–9. 36322849 10.1056/NEJMe2210975

[48] Wiley J, et al. Psilocybin for depression. N Engl J Med. 2021;385(9):860–2. 10.1056/NEJMc2108082 34437794

[49] Butler M, Jelen L, Rucker J. Expectancy in placebo-controlled trials of psychedelics: If so, so what? Psychopharmacology. 2022;239(10):3047–55. 36063208 10.1007/s00213-022-06221-6 PMC9481484

[50] Szucs D, Ioannidis JP. Sample size evolution in neuroimaging research: an evaluation of highly-cited studies (1990–2012) and of latest practices (2017–2018) in high-impact journals. Neuroimage. 2020;221:117164. 32679253 10.1016/j.neuroimage.2020.117164

[51] Kraehenmann R, et al. The mixed serotonin receptor agonist psilocybin reduces threat-induced modulation of amygdala connectivity. Neuroimage Clin. 2016;11:53–60. 26909323 10.1016/j.nicl.2015.08.009 PMC4732191

[52] Friston K. Ten ironic rules for non-statistical reviewers. Neuroimage. 2012;61(4):1300–10. 22521475 10.1016/j.neuroimage.2012.04.018

[53] Grimm O, et al. Psilocybin modulates functional connectivity of the amygdala during emotional face discrimination. Eur Neuropsychopharmacol. 2018;28(6):691–700. 29703645 10.1016/j.euroneuro.2018.03.016

[54] Mueller F, et al. Acute effects of LSD on amygdala activity during processing of fearful stimuli in healthy subjects. Transl Psychiatry. 2017;7(4):e1084. 28375205 10.1038/tp.2017.54 PMC5416695

[55] Arruda Sanchez T, et al. Emotion regulation effects of Ayahuasca in experienced subjects during implicit aversive stimulation: an fMRI study. J Ethnopharmacol. 2024. 10.1016/j.jep.2023.117430. 37979818 10.1016/j.jep.2023.117430

[56] Castelhano J, et al. The effects of tryptamine psychedelics in the brain: a meta-analysis of functional and review of molecular imaging studies. Front Pharmacol. 2021;12:739053. 34658876 10.3389/fphar.2021.739053 PMC8511767

[57] Murphy SE, et al. Effect of a single dose of citalopram on amygdala response to emotional faces. Br J Psychiatry. 2009;194(6):535–40. 19478294 10.1192/bjp.bp.108.056093 PMC2802527

[58] Godlewska BR, et al. Short-term SSRI treatment normalises amygdala hyperactivity in depressed patients. Psychol Med. 2012;42(12):2609–17. 22716999 10.1017/S0033291712000591 PMC3488813

[59] Tassone VK, et al. Amygdala biomarkers of treatment response in major depressive disorder: An fMRI systematic review of SSRI antidepressants. Psychiatry Res Neuroimaging. 2024;338:111777. 38183847 10.1016/j.pscychresns.2023.111777

[60] Disner SG, et al. Neural mechanisms of the cognitive model of depression. Nat Rev Neurosci. 2011;12(8):467–77. 21731066 10.1038/nrn3027

[61] Zavaliangos-Petropulu A, et al. Neuroimaging-derived biomarkers of the antidepressant effects of ketamine. Biol Psychiatry Cogn Neurosci Neuroimaging. 2023;8(4):361–86. 36775711 10.1016/j.bpsc.2022.11.005 PMC11483103

[62] Bush G, et al. Dorsal anterior cingulate cortex: a role in reward-based decision making. Proc Natl Acad Sci U S A. 2002;99(1):523–8. 11756669 10.1073/pnas.012470999 PMC117593

[63] Dedovic K, et al. Dorsal anterior cingulate cortex responses to repeated social evaluative feedback in young women with and without a history of depression. Front Behav Neurosci. 2016;10:64. 27065828 10.3389/fnbeh.2016.00064 PMC4815251

[64] Eisenberger NI, Lieberman MD, Williams KD. Does rejection hurt? An FMRI study of social exclusion. Science. 2003;302(5643):290–2. 14551436 10.1126/science.1089134

[65] Gasquoine PG. Localization of function in anterior cingulate cortex: from psychosurgery to functional neuroimaging. Neurosci Biobehav Rev. 2013;37(3):340–8. 23313645 10.1016/j.neubiorev.2013.01.002

[66] Rotge JY, et al. A meta-analysis of the anterior cingulate contribution to social pain. Soc Cogn Affect Neurosci. 2015;10(1):19–27. 25140048 10.1093/scan/nsu110 PMC4994851

[67] Palhano-Fontes F, et al. The psychedelic state induced by ayahuasca modulates the activity and connectivity of the default mode network. PLoS One. 2015;10(2): e0118143. 25693169 10.1371/journal.pone.0118143 PMC4334486

[68] Pasquini L, Palhano-Fontes F, Araujo DB. Subacute effects of the psychedelic ayahuasca on the salience and default mode networks. J Psychopharmacol (Oxf). 2020;34(6):623–35. 10.1177/0269881120909409 32255395

[69] Sampedro F, et al. Assessing the psychedelic “after-glow” in ayahuasca users: post-acute neurometabolic and functional connectivity changes are associated with enhanced mindfulness capacities. Int J Neuropsychopharmacol. 2017;20(9):698–711. 28525587 10.1093/ijnp/pyx036 PMC5581489

[70] Peng X, et al. Sub-regional anterior cingulate cortex functional connectivity revealed default network subsystem dysfunction in patients with major depressive disorder. Psychol Med. 2021;51(10):1687–95. 32151293 10.1017/S0033291720000434

[71] Müller F, et al. Altered network hub connectivity after acute LSD administration. Neuroimage Clin. 2018;18:694–701. 29560311 10.1016/j.nicl.2018.03.005 PMC5857492

[72] Carhart-Harris RL, et al. Neural correlates of the LSD experience revealed by multimodal neuroimaging. Proc Natl Acad Sci U S A. 2016;113(17):4853–8. 27071089 10.1073/pnas.1518377113 PMC4855588

[73] Tolle HM, et al. The unique neural signature of your trip: functional connectome fingerprints of subjective psilocybin experience. Netw Neurosci. 2024;8(1):203–25. 38562294 10.1162/netn_a_00349 PMC10898784

[74] Mertens LJ, et al. Therapeutic mechanisms of psilocybin: changes in amygdala and prefrontal functional connectivity during emotional processing after psilocybin for treatment-resistant depression. J Psychopharmacol. 2020;34(2):167–80. 31941394 10.1177/0269881119895520

[75] Kaiser RH, et al. Large-scale network dysfunction in major depressive disorder: A meta-analysis of resting-state functional connectivity. JAMA Psychiat. 2015;72(6):603–11. 10.1001/jamapsychiatry.2015.0071 PMC4456260 25785575

[76] Johnstone T, et al. Failure to regulate: counterproductive recruitment of top-down prefrontal-subcortical circuitry in major depression. J Neurosci. 2007;27(33):8877–84. 17699669 10.1523/JNEUROSCI.2063-07.2007 PMC6672169

[77] Wall BM, et al. Increased low-frequency brain responses to music after psilocybin therapy for depression. J Affect Disord. 2023;333:321–30. 37094657 10.1016/j.jad.2023.04.081

[78] Gazzaniga, M.S., R.B. Ivry, and G.R. Mangun. *Cognitive Neuroscience. The Biology of the Mind*. 4th ed., USA: W. W. Norton & Company, Inc; 2014.

[79] Carhart-Harris RL, et al. Functional connectivity measures after psilocybin inform a novel hypothesis of early psychosis. Schizophr Bull. 2013;39(6):1343–51. 23044373 10.1093/schbul/sbs117 PMC3796071

[80] Duerler P, et al. Psilocybin induces aberrant prediction error processing of tactile mismatch responses-a simultaneous EEG-FMRI study. Cereb Cortex. 2021;32(1):186–96. 34255821 10.1093/cercor/bhab202

[81] Clark L, Chamberlain SR, Sahakian BJ. Neurocognitive mechanisms in depression: implications for treatment. Annu Rev Neurosci. 2009;32:57–74. 19400725 10.1146/annurev.neuro.31.060407.125618

[82] Zhang FF, et al. Brain structure alterations in depression: Psychoradiological evidence. CNS Neurosci Ther. 2018;24(11):994–1003. 29508560 10.1111/cns.12835 PMC6489983

[83] Greicius MD, et al. Resting-state functional connectivity in major depression: abnormally increased contributions from subgenual cingulate cortex and thalamus. Biol Psychiatry. 2007;62(5):429–37. 17210143 10.1016/j.biopsych.2006.09.020 PMC2001244

[84] Li BJ, et al. A brain network model for depression: From symptom understanding to disease intervention. CNS Neurosci Ther. 2018;24(11):1004–19. 29931740 10.1111/cns.12998 PMC6490158

[85] Roseman L, et al. The effects of psilocybin and MDMA on between-network resting state functional connectivity in healthy volunteers. Front Hum Neurosci. 2014;8:204. 24904346 10.3389/fnhum.2014.00204 PMC4034428

[86] Copa D, et al. Predicting the outcome of psilocybin treatment for depression from baseline fMRI functional connectivity. J Affect Disord. 2024;353:60–9. 38423367 10.1016/j.jad.2024.02.089

[87] Schimmelpfennig J, et al. The role of the salience network in cognitive and affective deficits. Front Hum Neurosci. 2023;17:1133367. 37020493 10.3389/fnhum.2023.1133367 PMC10067884

[88] Preller KH, et al. Psilocybin induces time-dependent changes in global functional connectivity. Biol Psychiatry. 2020;88(2):197–207. 32111343 10.1016/j.biopsych.2019.12.027

[89] Koob GF, Volkow ND. Neurobiology of addiction: a neurocircuitry analysis. Lancet Psychiatry. 2016;3(8):760–73. 27475769 10.1016/S2215-0366(16)00104-8 PMC6135092

[90] Sullivan EV, et al. A selective insular perfusion deficit contributes to compromised salience network connectivity in recovering alcoholic men. Biol Psychiatry. 2013;74(7):547–55. 23587427 10.1016/j.biopsych.2013.02.026 PMC3766441

[91] Everitt BJ, Robbins TW. Neural systems of reinforcement for drug addiction: from actions to habits to compulsion. Nat Neurosci. 2005;8(11):1481–9. 16251991 10.1038/nn1579

[92] Goldstein RZ, Volkow ND. Dysfunction of the prefrontal cortex in addiction: neuroimaging findings and clinical implications. Nat Rev Neurosci. 2011;12(11):652–69. 22011681 10.1038/nrn3119 PMC3462342

[93] Garcia-Romeu A, Griffiths RR, Johnson MW. Psilocybin-occasioned mystical experiences in the treatment of tobacco addiction. Curr Drug Abuse Rev. 2015;7(3):157–64. 10.2174/1874473708666150107121331 PMC4342293 25563443

[94] Bogenschutz MP, et al. Percentage of heavy drinking days following psilocybin-assisted psychotherapy vs placebo in the treatment of adult patients with alcohol use disorder: a randomized clinical trial. JAMA Psychiat. 2022;79(10):953–62. 10.1001/jamapsychiatry.2022.2096 PMC9403854 36001306

[95] Stoliker D, et al. Neural mechanisms of psychedelic visual imagery. Mol Psychiatry. 2025;30(4):1259–66. 38862674 10.1038/s41380-024-02632-3 PMC11919690

[96] Power JD, et al. Functional network organization of the human brain. Neuron. 2011;72(4):665–78. 22099467 10.1016/j.neuron.2011.09.006 PMC3222858

[97] Raichle ME. The restless brain. Brain Connect. 2011;1(1):3–12. 22432951 10.1089/brain.2011.0019 PMC3621343

[98] Shen X, et al. Groupwise whole-brain parcellation from resting-state fMRI data for network node identification. Neuroimage. 2013;82:403–15. 23747961 10.1016/j.neuroimage.2013.05.081 PMC3759540

[99] Smith SM, et al. Correspondence of the brain’s functional architecture during activation and rest. Proc Natl Acad Sci U S A. 2009;106(31):13040–5. 19620724 10.1073/pnas.0905267106 PMC2722273

[100] Van Essen DC, et al. The WU-Minn Human Connectome Project: an overview. Neuroimage. 2013;80:62–79. 23684880 10.1016/j.neuroimage.2013.05.041 PMC3724347

[101] Yeo BTT, et al. The organization of the human cortex estimated by intrinsic functional connectivity. J Neurophysiol. 2011;106:1125–65. 21653723 10.1152/jn.00338.2011 PMC3174820

[102] Beckmann CF, et al. Investigations into resting-state connectivity using independent component analysis. Philos Trans R Soc Lond B Biol Sci. 2005;360(1457):1001–13. 16087444 10.1098/rstb.2005.1634 PMC1854918

[103] Martens MAG, et al. Resting state functional connectivity patterns as biomarkers of treatment response to escitalopram in patients with major depressive disorder. Psychopharmacol. 2022;239(11):3447–60. 10.1007/s00213-021-05915-7 PMC9584978 34477887

[104] Schrantee A, et al. Serotonin transporter occupancy by the SSRI citalopram predicts default-mode network connectivity. Eur Neuropsychopharmacol. 2018;28(10):1173–9. 30082141 10.1016/j.euroneuro.2018.07.099

[105] Shukuroglou M, et al. Changes in music-evoked emotion and ventral striatal functional connectivity after psilocybin therapy for depression. J Psychopharm. 2023;37(1):70–9. 10.1177/02698811221125354. 10.1177/02698811221125354 PMC9834320 36433778

[106] Varley TF, et al. Serotonergic psychedelics LSD & psilocybin increase the fractal dimension of cortical brain activity in spatial and temporal domains. NeuroImage. 2020;220:117049. 32619708 10.1016/j.neuroimage.2020.117049

[107] Mortaheb S, et al. Dynamic Functional Hyperconnectivity after Psilocybin Intake is Primarily Associated with Oceanic Boundlessness. Biol Psychiatry Cogn Neurosci Neuroimaging. 2024;9(7):681–92. 38588855 10.1016/j.bpsc.2024.04.001

[108] Stroliker D, et al. Neural mechanisms of psychedelic visual imagery. Mol Psychiatry. 2025;30(4):1259–66. 38862674 10.1038/s41380-024-02632-3 PMC11919690

